# Publisher Correction: Laser spectroscopy of the $^2{\mathrm{S}}_{1/2}{-}^2{\mathrm{P}}_{{1}/2}$, $^2{\mathrm{P}}_{3/2}$ transitions in stored and cooled relativistic ${\text{C}}^{3+}$ ions

**DOI:** 10.1038/s41598-021-97511-0

**Published:** 2021-08-30

**Authors:** D. Winzen, V. Hannen, M. Bussmann, A. Buß, C. Egelkamp, L. Eidam, Z. Huang, D. Kiefer, S. Klammes, Th. Kühl, M. Loeser, X. Ma, W. Nörtershäuser, H.-W. Ortjohann, R. Sánchez, M. Siebold, Th. Stöhlker, J. Ullmann, J. Vollbrecht, Th. Walther, H. Wang, Ch. Weinheimer, D. F. A. Winters

**Affiliations:** 1 Institute for Nuclear Physics, University of Münster, 48149 Münster, Germany; 2 Helmholtz Center Dresden-Rossendorf, 01328 Dresden, Germany; 3 Institute for Accelerator Science and Electromagnetic Fields, Technical University of Darmstadt, 64289 Darmstadt, Germany; 4 Institute of Modern Physics, Chinese Academy of Sciences, Lanzhou, 730000 China; 5 Institute for Applied Physics, Technical University of Darmstadt, 64289 Darmstadt, Germany; 6 GSI Helmholtz Center for Heavy Ion Research, 64291 Darmstadt, Germany; 7 Helmholtz Institute Jena, 07743 Jena, Germany; 8 Institute of Physics, University of Mainz, 55099 Mainz, Germany; 9 Institute for Nuclear Physics, Technical University of Darmstadt, 64289 Darmstadt, Germany; 10 Institute for Optics and Quantum Electronics, University of Jena, 07743 Jena, Germany; 11 Helmholtz Research Academy Hesse for FAIR (HFHF), GSI Helmholtz Centre for Heavy Ion Research, Campus Darmstadt, Darmstadt, Germany

Correction to: *Scientific Reports* 10.1038/s41598-021-88926-w, published online 30 April 2021

The original PDF version of this Article contained a typographical error in the title of the paper, where “C^3+^” was incorrectly given as “C_3+_”

The original Article has been corrected.

